# New Appliances and Things Medical

**Published:** 1903-07-04

**Authors:** 


### NEW APPLIANCES AND THINGS MEDICAL.

[We shall be glad to receive, at our Office, 28 & 29 Southampton Street Strand, London, W.C., from the manufacturers, specimens of all new preparations and appliances which may be brought out from time to time.]

### REVERSIBLE COMMODE AND BIDET.

(GEORGE JENNINGS, LIMITED, LAMBETH PALACE ROAD, LONDON, S.E.)

THE firm of George Jennings, Limited, have recently patented a very ingenious and useful form of commode and bidet combined. The apparatus is manufactured in earthenware and in enamelled iron, and may be obtained in both oval and circular form. The portion which is to be used as a commode is supplied with a handle and a closely-fitting cover, which can be rendered air-tight by filling a fluted rim with water. The commode portion fits into the stand, which elevates it to a convenient height for sitting upon, while the stand itself, if not used for this purpose, can be turned over and employed as a bidet, foot-bath, or lavatory basin. The great advantages of this ingenious combination are:—1. The sanitary and cleanly character of the commode and stand; the whole apparatus can be readily cleaned and rendered aseptic. 2. Economy in space; this is a valuable point for use on board yachts, and in bungalows and flats.

